# Integrated overview of stramenopile ecology, taxonomy, and heterotrophic origin

**DOI:** 10.1093/ismejo/wrae150

**Published:** 2024-07-30

**Authors:** Dagmar Jirsová, Jeremy G Wideman

**Affiliations:** Center for Mechanisms of Evolution, Biodesign Institute, School of Life Sciences, Arizona State University, 1001 S McAllister Avenue, Tempe, Arizona, 85287-7701, United States; Institute of Parasitology, Biology Centre, Czech Academy of Sciences, Branišovská 31, České Budějovice 37005, Czech Republic; Center for Mechanisms of Evolution, Biodesign Institute, School of Life Sciences, Arizona State University, 1001 S McAllister Avenue, Tempe, Arizona, 85287-7701, United States

**Keywords:** stramenopiles, heterotrophic flagellates, plastid evolution, chromalveolate hypothesis, rhodoplex hypothesis, protistology, microbial ecology and evolution

## Abstract

Stramenopiles represent a significant proportion of aquatic and terrestrial biota. Most biologists can name a few, but these are limited to the phototrophic (e.g. diatoms and kelp) or parasitic species (e.g. oomycetes, *Blastocystis*), with free-living heterotrophs largely overlooked. Though our attention is slowly turning towards heterotrophs, we have only a limited understanding of their biology due to a lack of cultured models. Recent metagenomic and single-cell investigations have revealed the species richness and ecological importance of stramenopiles—especially heterotrophs. However, our lack of knowledge of the cell biology and behaviour of these organisms leads to our inability to match species to their particular ecological functions. Because photosynthetic stramenopiles are studied independently of their heterotrophic relatives, they are often treated separately in the literature. Here, we present stramenopiles as a unified group with shared synapomorphies and evolutionary history. We introduce the main lineages, describe their important biological and ecological traits, and provide a concise update on the origin of the ochrophyte plastid. We highlight the crucial role of heterotrophs and mixotrophs in our understanding of stramenopiles with the goal of inspiring future investigations in taxonomy and life history. To understand each of the many diversifications within stramenopiles—towards autotrophy, osmotrophy, or parasitism—we must understand the ancestral heterotrophic flagellate from which they each evolved. We hope the following will serve as a primer for new stramenopile researchers or as an integrative refresher to those already in the field.

## Graphical Abstract

**Graphical Abstract:**
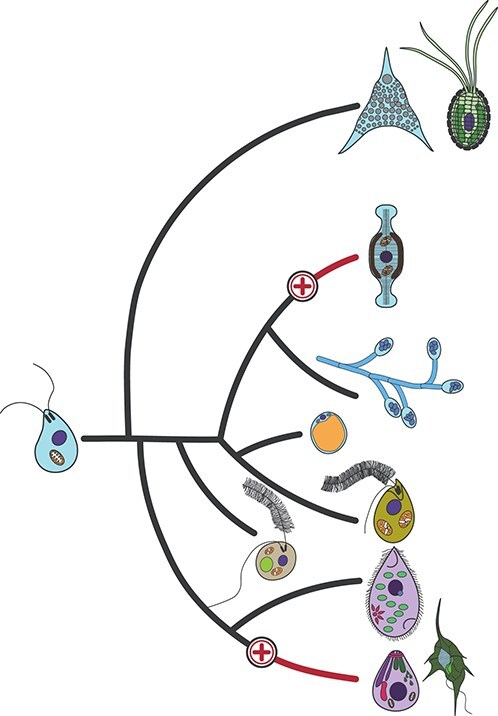

## Introduction

Stramenopiles are mostly microbial eukaryotes, and can be found in virtually all habitats, including freshwater and soil [1, 2], oceans [3, 4], arctic waters [4, 5], the deep sea [6, 7], and deserts [7–9]. Most people are familiar with stramenopiles, even if they have never heard the word. Kelp are stramenopiles—so are diatoms. The Irish Potato Famine was caused by the blight-causing oomycete *Phytophthora infestans*, a filamentous fungus-like stramenopile [10]. Many people carry one of the subtypes of the intestinal commensal *Blastocystis*, a heterotrophic stramenopile that forms resilient cysts [11].

Stramenopiles are ecologically and evolutionarily important. From an ecological perspective, stramenopiles contribute greatly to carbon and nutrient cycling. Phototrophic stramenopiles, diatoms in particular, produce almost ~40% of global ocean-derived oxygen, which is comparable to the Amazonian rain forest [12]. Heterotrophic stramenopiles keep the carbon and mineral cycles churning as voracious consumers of bacteria and other microbes [13, 14]. Some stramenopiles are supplementing their photosynthetic capabilities through phagotrophy or osmotrophy, exhibiting mixotrophic life strategy [15–17]. Their position in a food chain is more complex since the ability to function as a primary producer or consumer is difficult to evaluate. Thus, phototrophic, mixotrophic, and heterotrophic groups likely play important roles in most ecosystems [2, 3, 18–20]. However, our ecological knowledge of heterotrophs is mostly limited to 18S rRNA gene amplicon sequencing (e.g. small ribosomal subunit V4 and V9 regions), which are effective [18, 19], though heavily biased [21–23], and uninformative when recovering unsampled or under investigated lineages [24]. As a recent report suggests, “One of the main issues [preventing] us from obtaining a detailed assessment on the ecology of heterotrophic flagellates […] is the lack of cultured strains that effectively represent the dominant heterotrophic flagellate species in the ocean” [18]. Therefore, traditional natural history, taxonomy, cultivation, and whole genome sequencing projects are desperately needed to improve our ecological understanding of heterotrophic flagellated stramenopiles, including their contributions to food webs and the roles they play within diverse ecosystems.

From an evolutionary perspective, stramenopiles have colonized and adapted to many unique environments and are key to understanding cell biological transitions to phototrophy, mixotrophy, osmotrophy, and parasitism. Again, though we know substantially more about phototrophs and parasites, we know very little about the free-living heterotrophic flagellates [18], from which phototrophs (including mixotrophs) and parasites emerged (e.g. [16, 25]). As eukaryotic microbial dark matter comes into light, we must develop strategies to culture and investigate newfound heterotrophic lineages that occupy important phylogenetic positions on the stramenopile tree of life. In-depth investigations of these lineages will be critical to understanding both their ecological and biochemical importance [26] and their ancestor’s capacity to diversify and occupy open niches.

**Figure 1:**
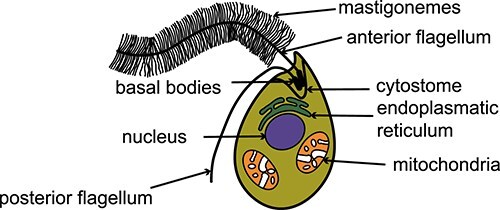
A representation of a typical heterotrophic stramenopile. Stramenopiles have two unequal flagella; the anterior is equipped with two rows of tripartite tubular hairs called mastigonemes. Some taxa have a specific phagocytic apparatus that acts like a “cell mouth” called a cytostome. Early phototrophic stramenopiles were likely similar to this representation but with an addition of a complex red plastid [209].

Stramenopiles were originally called heterokonts, with both names deriving from shared morphological characters. Heterokont simply means “two different flagella,” and stramenopile (stramen = straw; piles = hair) derives from the hair-like tripartite tubular protrusions (mastigonemes) (Fig. 1). Mastigonemes have been recognized as characteristic of stramenopile cells for many decades [27–30]. Although they are lost in some clades (e.g. diatoms [31], *Cafileria marina* [32]) and present in some nonstramenopiles (e.g. cryptophytes [33], bodonids [34]), their tripartite structure and placement on both sides of the anterior flagellum make them unique to stramenopiles. In some stramenopiles, mastigonemes are not very obvious features but remain present for motile lifecycle stages (e.g. zoospores of kelp [35], oomycetes [36], and labyrinthulomycetes [37]). So far, we know that mastigonemes enhance flagellar propulsive force by increasing surface area and also act as mechanosensors [38, 39], though other potential functions remain enigmatic [40]. Genomics and cell biological investigations of stramenopiles have revealed a few more shared characters. Stramenopiles universally lack mitochondrial-encoded threonine tRNA [41], contain a fused version of the glycolysis enzymes glyceraldehyde-3-phosphate dehydrogenase and triosephosphate isomerase [42, 43], and have relocalized the ATP-yielding part of glycolysis from the cytosol to the mitochondria [43, 44]. More clade-defining features are likely present, but we lack the model systems to investigate the vast diversity of stramenopiles.

In this review, we will introduce the broad diversity of stramenopiles and highlight important aspects of their morphology, ecology, and evolutionary history. Fortunately, the interest in free-living heterotrophic stramenopiles is growing [3, 32, 45–48], which only highlights the need to further investigate this group.

## Stramenopiles are the “S” in SAR

**Figure 2:**
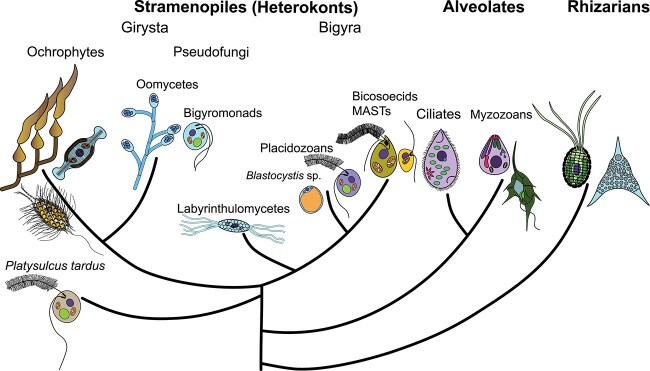
A simplified phylogeny of the SAR clade, with a focus on stramenopiles. Rhizarians and alveolates are distant relatives of stramenopiles. Within stramenopiles, there are two major well-supported groups: Bigyra and Gyrista. Whereas Bigyra consist of heterotrophs only, Gyrista include the only plastid possessing group—Ochrophyta. Within the Bigyra and Gyrista, branching orders are still being worked out as new lineages and species of stramenopiles are consistently being discovered (e.g. MArine STrameonpiles (MASTs) [4] and *Platysulcus tardus* [150]) or the phylogenetic position of already known species is being reclassified by more robust genomic and transcriptomic data (e.g. *P. flagellatus* [48, 210], *﻿A. sol* [45, 48]).

Stramenopiles, Alveolates, and Rhizarians form the monophyletic group called SAR [49] (Fig. 2). A fairly clear branching order of the three groups has emerged with rhizarians at the base of the group and alveolates + stramenopiles as sister clades to one another [50–52], although some studies place rhizarians as a sister group to alveolates and stramenopiles at the base (e.g. [53, 54]). Apart from the fact that they always branch together in gene-based phylogenetic trees, these three groups do not seem to have much in common (though some molecular synapomorphies have been identified, e.g. [55]). Though there are no apparent cellular or morphological similarities observed between SAR groups, it has been clear for several decades that stramenopiles form a monophyletic group. This early conjecture was based on their unique flagellar traits described above [56].

Stramenopiles comprise two usually well-supported clades, the Bigyra and Gyrista [27, 57–60] (Fig. 2), although lately, the monophyly of Bigyra has been shaken [61]. Gyrista includes the predominantly osmotrophic pseudofungi and the photosynthetic ochrophytes, which consist of some lineages known only from sequence data called Marine OCHrophytes (MOCHs) [4, 48], encompassing marine as well as freshwater and terrestrial ochrophytes [48]. Bigyra are strictly heterotrophic and include bikosians, labyrinthulomycetes, and placidozoans [57, 59, 60, 62, 63]. Both Bigyra and Gyrista contain several lineages that are not well characterized, many of which are referred to as MArine Stramenopiles (MASTs) [4], even if some are not marine [4, 64]. In the following, we outline what is known about the major groups of stramenopiles and highlight avenues for future research.

## Ochrophytes are the only photosynthetic group of stramenopiles

Compared to heterotrophic stramenopiles, ochrophytes have enjoyed significant scientific attention. They represent a clade of extremely diverse eukaryotic autotrophs [65, 66], which includes photosynthetic nano- and pico- algae, giant kelps, and even derived heterotrophs that secondarily lost their photosynthetic capabilities [45, 48, 67–71]. One of the peculiar features of many ochrophytes is their ability to be mixotrophs (e.g. chrysophytes [15], *Microchloropsis* sp. [16], Parmales/Bolidophyceae [17]). Mixotrophy occurs in many plastid-bearing lineages (e.g. green algae Prasinophyta [72], dinoflagellate *Karlodinium veneficum* [73], haptophytes such as *Phaeocystis* [74]) in which autotrophs maintain heterotrophy as a compensation for specific nutrient deficiencies [75]. As important primary producers and secondary consumers, mixotrophs have complex roles in food webs [76, 77], though they are excluded from most ecological models [76].

Ochrophytes have a specific combination of photosynthetic pigments that gives their plastid its brown colour (chlorophyll *c,* chlorophyll *a,* and the accessory pigment—fucoxanthin) [78]. Exceptions include Xanthophyceae and Eustigmatophyceae (yellow-green algae) as they have only chlorophyll *a* and β-carotenes [79, 80]. The best studied ochrophytes are the diatoms (Bacillariophyceae). These microalgae are known for their spectacularly shaped silica-based shells (aka frustules). Their significant impact on oxygen production [12] and carbon fixation [81] makes them the most successful ocean phototrophs. Their natural production of ﻿triacylglycerols and fucoxanthin makes them a potential candidates for the biofuel industry [82], whereas their silica shells are used for pest control [83] and as environmental fertilizers [84]. Diatoms are also one of the few genetically tractable stramenopiles [85, 86].

Though diatoms are the most scientifically explored of all stramenopiles, the most famous ochrophytes are the kelp. Kelp dominate cold ocean areas of low water motion, with kelp forests providing interesting ocean habitats [87, 88]. Kelps (Phaeophyceae, aka brown algae) have the typical brown colouring of ochrophytes and their characteristic stramenopile sexual zoospore stage, which is complete with mastigonemes that have been noted for many decades [89]. As primary producers, kelp and diatoms are essential part of food webs [90, 91], and any environmental changes will affect the species representation causing a domino effect in other ecosystem tiers [88, 92].

Besides diatoms, other highly abundant and omnipresent ochrophytes include the chrysophytes (aka golden algae) [3, 18, 93, 94]. Chrysophytes are mostly solitary biflagellate algae but can form colonies, and similar to diatoms, some species have silica shells (lorica) [15]. Chrysophytes encompass wide variation in their nutritional modes, from plastid-bearing phototrophs (e.g.*﻿ Ochromonas* sp. and *Chromulina* sp. [94]), to photosynthetic mixotrophs (e.g. some *Ochromonas* species [95]), bacteriovorus phago-mixotrophs (e.g. *Ochromonas* [95, 96]), and even heterotrophs (e.g. *Spumella* sp. and *Spumella*-like sp. [97]), which no longer photosynthesize but retain a leucoplast—a colourless plastid. Mixotrophy is not exclusive to chrysophytes but also occurs in other ochrophytes like diatoms [98, 99] and coccolithophores [100, 101]. Heterotrophic nutrient uptake in mixotrophy can occur through phagotrophy or osmotrophy, with osmotrophy being common when cells have physical barriers like silica shells [98–101]. A complete shift to osmotrophy can lead to plastid simplification and loss of photosynthetic ability, as seen in *Nitzschia putrida* [102]. Mixotrophy may offer a selective advantage over phototrophy or heterotrophy alone [99].

One reason chrysophytes might have maintained mixotrophy is their relatively poor capacities to fix carbon, assimilate nitrogen [103–105], and uptake phosphorus [106, 107]. Additionally, ﻿chrysophytes cannot make thiamine (B1 vitamin) [25], which leads to a dependency on specific members of the microbial community [108]. The mixotrophy amongst algae seems to be more prevalent than we originally thought and was adopted or maintained multiple times in different lineages [109, 110], perhaps rightfully so, as it is the transitional stage between heterotrophy and obligate autotrophy. Therefore, mixotrophy is an important part in plastid acquisition and its genomic and metabolic integration (see Ochrophyte plastid: count the gains, not the losses section).

As mixotrophs, chrysophytes are metabolically and genetically diverse [25, 95]. With the plastid comes advantages and trade-offs as the new organelle is adopted into a functioning eukaryotic cell; some metabolic pathways show significant differences compared to heterotrophs. The most prominent alterations can be seen in the heme pathway [70, 111], isoprenoid [67], and fatty acid synthesis [112, 113]. In addition to their significant ecological impact, this makes the chrysophytes great model organisms for studying plastid genome reduction and metabolic evolution [69]. Besides these well-described taxa, 10 MOCH clades are scattered across ochrophytes, which are known exclusively from sequence data [4, 48].

### Ochrophyte plastid: count the gains, not the losses

**Figure 3:**
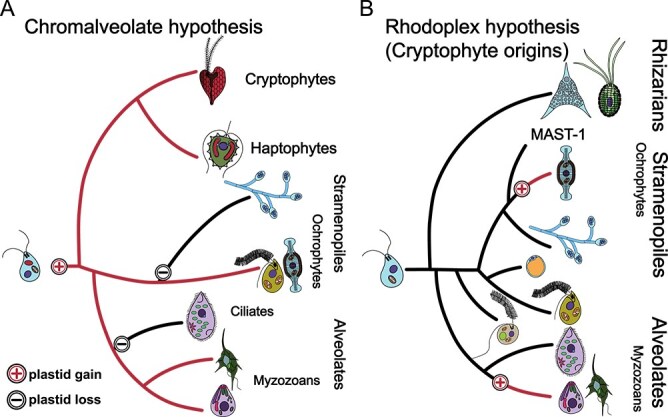
The origin of complex plastids. (A) The chromalveolate hypothesis. The originally proposed chromalveolate hypothesis [118] suggests that all CASH plastids arose from a single secondary endosymbiotic event. Complex plastids were thought to be lost in a few known heterotrophic lineages related to complex plastid-bearing lineages. The chromalveolate hypothesis was supported by early phylogenies that erroneously grouped the Cryptophyta, Alveolata, Stramenopila, and Haptophyta (CASH lineages) together based on striking similarities of their plastids genes. However, with new data, for this hypothesis to remain in contention, organellar loss must be an easier accomplishment than once thought. (B) The rhodoplex hypothesis. As more heterotrophic lineages were shown to be more closely related to individual CASH lineages than the CASH lineages were to one another, more and more losses were required for the chromalveolate hypothesis to remain true. Instead, data now strongly suggest that the ancestor of stramenopiles (and SAR) was heterotrophic. Thus, instead of a single plastid gain followed by multiple losses, four serial gains of complex plastids have been reported explaining plastid possession in the CASH lineages. In the stramenopiles, recent studies strongly suggest that the plastid of ochrophytes was obtained through a tertiary endosymbioses of a cryptophyte [147].

Although ochrophytes are the most studied group of stramenopiles, their plastid’s origin story remains incomplete. The group evolved over 500 million years ago [114], but the evolutionary origin of its plastid is only just coming to light. Although it is undisputed that the ochrophyte plastid has a red algal origin [115–117], there are two predominating theories of how this arose (Fig. 3). In the first scenario, the ancestor of the SAR lineage had already acquired a secondary red plastid, which was subsequently lost in all related heterotrophic lineages (Fig. 3A). The second scenario starts with a heterotrophic ancestor and requires multiple plastid acquisitions across the evolutionary tree, including one in stramenopiles (Fig. 3B).

#### How many losses are too many losses?

The first hypothesis—the “chromalveolate hypothesis” [118]—posits the ancestor of a now-defunct photosynthetic clade called chromalveolates (stramenopiles, alveolates, cryptophytes, and haptophytes) had a secondary red algal-derived plastid [119, 120]. The chromalveolate hypothesis presumes that all heterotrophic lineages related to any of these groups independently lost their plastid. When this hypothesis was first proposed, only a few related heterotrophic SAR lineages were known (e.g. ciliates—a group of alveolates covered with hair-like cilia, oomycetes, goniomonads—aplastidial cryptists), suggesting that multiple plastid losses were the most feasible scenario. Shared characters between chromalveolates such as the presence of chlorophyll *c* [78, 118] and robust plastid phylogenies [121–123] supported the monophyly of this group. Additionally, some evidence of the historical plastid was found in heterotrophic lineages (e.g. oomycetes [124], labyrinthulomycetes [125]). Unfortunately, these early analyses had suffered from a lack data for known groups. They focused nearly exclusively on phototrophs, and conclusions were based largely on plastid genes [126], or very small datasets in which signal was indistinguishable from noise [127]. Thus, although the multiple plastid losses proposed by the chromalveolate hypothesis seemed likely at the time, the rarity of organellar loss was not yet fully appreciated or understood.

Now we know that once an endosymbiotic organelle is acquired, its complete loss is exceedingly rare—although reduction and loss of organellar functions and even genomes are quite common (e.g. highly reduced mitochondria in anaerobic lineages [128, 129]). In the case of mitochondria, the only confirmed loss of the organelle occurred in the anaerobe *Monocercomonoides* [130]. In parasitic lineages, organellar reduction is not surprising, as most simplify their metabolism because of capitalizing on host-derived substrates [131–133]. Despite the commonness of functional simplification, we know of only two examples of complete plastid loss in parasites: the intestinal apicomplexan *Cryptosporidium* [134] and the parasitic dinoflagellate *Hematodinium* sp. [135]. An example of a putative loss in a free-living lineage has only recently come to light in the Picozoa, which branch sister to red algae and *Rhodelphis* [136]. Finally, recently discovered plastid losses have been identified in the ochrophyte lineage (e.g. the flagellate ﻿*Picophagus flagellatus* and the heliozoan *﻿Actinophrys sol* [48]). Although these cases demonstrate the possibility of total organelle loss, it speaks to the rarity of the event.

In acknowledgement of the rarity of plastid loss, the chromalveolate hypothesis became less feasible. Furthermore, as rhizarians were shown to be related to stramenopiles and alveolates, and cryptophytes and haptophytes were revealed to branch only distantly to the SAR lineage [137, 138], more and more plastid losses were required for the chromalveolate hypothesis to remain true. As more heterotrophs were found, a growing number of aplastidial lineages were shown to be more closely related to individual lineages within chromalveolates than the photosynthetic chromalveolates were to each other. These discoveries resulted in the eventual downfall of the chromalveolate hypothesis. Thus, the field concluded that plastid gain is not as rare as we once thought, and it is now accepted that multiple plastid gains is the more likely scenario. For example, considering only alveolates and stramenopiles, the minimum number of plastid losses required for the chromalveolate hypothesis to remain in contention is between five and seven [46], whereas only two red-algal derived plastid gains are required within the entire SAR clade.

#### How can multiple gains be a realistic explanation?

With the chromalveolate hypothesis disproven, a multiple origins hypothesis became necessary. Remember, the chromalveolate [now sometimes referred to as “CASH” (Cryptophytes, Alveolates, Stramenopiles, and Haptophytes)] lineages of plastids all contain chlorophyll *c* and are very closely related based on plastid phylogenies [121, 139]. These data suggest that their plastid came either from multiple secondary endosymbioses of very closely related red algae or a series of higher-order endosymbioses that occurred between CASH groups. Again, the first option seems simpler. However, the secondary plastids of cryptophytes, stramenopiles, and haptophytes all share a phylogenetically related and specialized protein sorting machinery derived from the ER-Associated protein Degradation system (ERAD) of red algae [140–142]. This machinery is called SELMA (Symbiont-specific ERAD-Like MAchinery) and is responsible for transporting proteins into and through the second (from the outside) plastid membrane [143, 144]. These data strongly suggest that CASH plastids all have a common endosymbiotic origin. But how can that be if multiple endosymbioses are necessary?

The rhodoplex hypothesis starts with a heterotrophic ancestor and introduces a new element of complexity. Instead of multiple secondary origins, only a single secondary endosymbiotic event occurred, and other red algal-derived plastids were acquired by tertiary or even quaternary endosymbioses [139, 145, 146]. The rhodoplex hypothesis was originally proposed as agnostic towards which lineage contained the original secondary endosymbiosis. Recent papers have suggested that molecular timescales and phylogenetic analyses are still compatible with multiple downstream scenarios; however, the original secondary endosymbiosis has likely been narrowed to the cryptophyte lineage [117, 147, 148]. These findings indicate that ochrophyte, alveolate, and haptophyte algae could have been derived from tertiary endosymbioses of cryptophytes or even more complex quaternary endosymbioses [117]. Additionally, modern cryptophytes have diverged quite recently (~200–300 MYA [147]); thus, most of the available evidence suggests that ochrophytes acquired their plastid via tertiary endosymbioses of an extinct lineage of cryptophytes [66, 147, 142] plausibly via kleptoplasty as the nucleomorph is missing [149].

#### Towards an understanding of the ancestor of ochrophytes

With the fall of the chromalveolate hypothesis came an understanding that plastid loss has not happened in the stramenopile heterotrophic lineages. Thus, stramenopiles are ancestrally heterotrophic. So far, no evidence of historical plastid presence has ever been demonstrated in any plastid-lacking stramenopile lineage, with the above-mentioned exceptions of ﻿*P. flagellates* and *﻿A. sol* [48]. No evidence of historical plastid loss remains for the major lineage comprising pseudofungi and bigyromonads [46], nor any bigyran, nor the deep-branching *Platysulcus tardus* [150, 59]); therefore, we can conclude that the ancestral stramenopile was a free-living heterotroph.

## Oomycetes: pseudofungi with heterotrophic flagellated relatives, the ﻿bigyromonads

Unlike autotrophic stramenopiles that cluster into a single group, heterotrophic stramenopiles can be found across several major stramenopile clades [50, 57]. Sister to ochrophytes are the pseudofungi (Oomycetes) and bigyromonads [57, 151], which includes saprotrophs [152, 153], though the best known and studied representatives are the parasitic oomycetes (aka water moulds). The lineages closely related to oomycetes are nonparasitic, but maintain an osmotrophic, fungi-like lifestyle (e.g. *Hyphochytrium* [27, 154]), Although they look like fungi, pseudofungi have zoospores with two unequal flagella and mastigonemes as a part of the life cycle [36]. Oomycetes can be found in aquatic and terrestrial habitats, have a filamentous structure, and because of this, were historically considered as a basal group of “true” fungi [155]. The similarities between fungi and pseudofungi, including their cell structure and osmotrophic lifestyle, were greatly affected by horizontal gene transfers (HGTs) from bacteria, animals [156], and fungi [157]. In particular, many genes associated with parasitism were transferred, including those encoding enzymes that break down polysaccharides and associated transporters that enable a parasite to feed on nutrients obtained from the host [158]. Historically, oomycetes provided key evidence for the chromalveolate hypothesis as their genomes contained genes of “red algal” origin [124]. However, the red algal ancestry of these genes was disproven by analysing a larger data set with different methods [120, 127].

Sister to these filamentous lineages [46], and somewhat overlooked due to the general lack of data, are the bigyromonads. This group of heterotrophic phagotrophs include the eukaryovore *Develorapax marinus* [159], the bacteriovores *Developayella elegans* [160], and *Mediocremonas mediterraneus* [161]. With bigyromonads as sisters to the oomycetes, investigations of these free-living heterotrophic flagellated stramenopiles are required to better understand transitions to osmotrophy, parasitism, and a filamentous cellular structure.

## Bigyra are a melange of mostly heterotrophic flagellates

In comparison to Gyrista, we know substantially less about Bigyra. Most known taxa are free-living heterotrophs, though some parasitism/commensalism has evolved in the opalinids and labyrinthulomycetes (e.g. *Blastocystis* sp. [162], *Aplanochytrium kerguelensis* [163]). Beyond *Blastocystis*, the best studied groups are the labyrinthulomycetes and bicosoecids (discussed below). Most of the assorted unidentified stramenopiles are known almost completely by sequence data and are called MArine STramenopiles (MASTs) [4, 64, 93, 164, 165]. Over the past years, the number of newly identified organisms in these groups is constantly rising due to environmental DNA surveys. Environmental studies (e.g., *Tara* Oceans expeditions) confirm this richness and estimate thousands of unidentified species belonging to MAST clades [4, 23, 59, 166]. Unfortunately, for the vast majority of MASTs, we lack microscopic observation and only have 18S or single-cell omics data, making it almost impossible to pair an organism with its sequence data [59, 164, 166, 167]. As MASTs are being cultured, morphologically described, and formally classified, the moniker will slowly disappear (e.g. MAST-3 are called Nanomonadea [168], and MAST-4 and 6 are called Eogyrea [59, 169]). To date, only a few MASTs have been phylogenetically placed [60, 164], with many more awaiting rediscovery and further investigation.

Branching outside both the Bigyra and Gyrista is a single species, *Platysulcus tardus*, an enigmatic heterotrophic flagellate [150, 59]. This stramenopile shares some key stramenopile features—two uneven flagella, the anterior equipped with mastigonemes, microtubular root, and its mitochondria exhibit tubular cristae [150]—but this species also has a few unique characteristics (﻿e.g. a flat vesicle surrounds the cytoplasm containing a nucleus, mitochondria, and microbodies). As we explore the enigmatic heterotrophs, we will no doubt begin to understand their diversified morphology and ecological strategies.

### Labyrinthulomycetes comprise slimy heterotrophs, flagellates, and some surprises

Bigyra also includes a somewhat well-studied group of filamentous heterotrophs called Labyrinthulomycetes. They are named for their most famous members, which create labyrinthine slime nets or “slime tubes.” These tubes create a network on which cells slither and absorb nutrients [170]. Most described species are marine saprotrophs, though some parasites of algae and animals also exist [171–174]. Beyond the more famous members for which they are named, labyrinthulomycetes also contain phagotrophs, including species with amoeboid stages that nutritionally exploit single-cell algae [175] and mixotrophic taxa with endosymbiotic green algae [176]. In the same way as oomycetes, labyrinthulomycetes have motile spores with two flagella, the anterior equipped with mastigonemes [177]. Though the group is mostly known for being marine, freshwater and terrestrial members are also being identified [177–179]. For a time, the labyrinthulomycetes entered the Chromalveolate debate [170] because of their abilities to produce omega-3 polyunsaturated fatty acids using a plastid-like desaturase [125]. However, this biochemical anomaly was later attributed to HGT from marine bacteria [180]. The biochemistry of this group has become industrially relevant, and as a result, a few species have been recently developed as genetically tractable models (e.g. *Parietichytrium* sp. and *Thraustochytrium* sp. [181]). This lineage is only just beginning to receive the attention it deserves. Similar to ochrophytes and oomycetes, an ancestral heterotrophic flagellated stramenopile was inevitably the predecessor to this clade, and once again, a better understanding of the transition to a fungal-like form requires deeper investigations into heterotrophic flagellates.

### Bikosia are key heterotrophs in aquatic ecosystems

As prime bacterial consumers, bikosians are an essential part of food webs and nutrient cycling through remineralization [13, 182, 183]. About 20 well-defined species of bicosoecids have been described with some affiliations to MASTs [57, 164, 184]. Despite their previous assignment near photosynthetic ochrophytes based on their ultrastructure [185, 186], phylogenetic analyses consistently show that bicosoecids are definitively bigyrids [187], with Opalinata (Slopalinida and *Blastocystis*) and placidideans branching nearby [57, 59, 60]. Bicosoecids share with chrysophytes a putatively ancestral-like feeding basket with a unique microtubular organization [169, 187] and also the heterotrophic/phagotrophic life strategy [95, 188]. The bicosoecid flagellar apparatus has a unique microtubular root (R3) that loops around the cell and creates additional physical support [184].

Bicosoecids include one of the best studied free-living heterotrophic stramenopiles, *Cafeteria roenbergensis* [189], as well as several more recently described species (e.g. new species in *Cafeteria* [9], *Cafileria marina* [32], and *Bilabrum latius* [190]). *Cafeteria* is a common and highly abundant part of marine plankton with many well-identified species [7, 166]. Its mitochondrial genome was the first to be assembled from Bikosia [191], and drafts of nuclear genomes were recently published [192]. Even prior to obtaining genomic data, *C. roenbergensis* was a model for studying bacterial grazing [193–195] and viral infection for the giant marine DNA virus CroV [196, 197]. Thus, *Cafeteria* should be an ideal model organism to study heterotrophic stramenopiles. However, we still lack basic information about its cell biology, further necessitating the development of more genetically tractable model organisms in the stramenopile lineage.

### Placidozoa span the normal to the bizarre

Placidozoans were first introduced in 2013 [168] as a closely related group to Bikosia [59, 60, 168], consisting of several groups of heterotrophic flagellates from the MAST-3 or Nanomonadea (including the parasitic *Solenicola setigera* [198] and the marine uniflagellated heterotroph *Incisomonas marina* [168]) as well as strictly intestinal protists in Opalinata. Placididea were recognized as a separate group in 2002 [199] with two taxa *Wobblia lunata* [62] and *Placidia cafeteriopsis* [199], but the number of described taxa is growing (e.g. [200]). Placidideans are often isolated from the deep sea halophilic environments, but in cultured conditions, they can tolerate lower levels of salinity [201, 202]. Their kidney-shaped cells, ultrastructural features (presence of mastigonemes on anterior flagellum), and movement all resemble *Cafeteria* sp. [201]. However, despite continuous effort, unlike *Cafeteria*, they are difficult to cultivate and remain mostly known from sequence data [60].

Opalinids are rather unusual protists, as all known taxa are intestinal commensals. Their cells are large (up to 3 mm) with multiple nuclei (2–200) and flagella or shorter cilia [203]. It is not surprising that based on these ciliate-like features, they were originally misclassified as ciliates [204]. They are mostly known to inhabit the cloacae of amphibians and lizards (e.g. *Opalina* sp. [205, 206]). Due to their obscure life style, opalinids are very under-sampled and their inner phylogenetic relationships are not resolved [207]. The related vertebrate intestinal commensal *Blastocystis* sp. [11, 162] is unlike its more decorated cousins. Its cells are spherical with a large central vacuole and one or two nuclei and lack flagella completely [208]. With extreme morphological plasticity and lifecycle complexity, this group can reveal how radiations can fill several available niches; however, due to the lack of a free-living heterotrophic model, our understanding is largely limited to phylogenies and comparative morphology.

## Conclusion

Why should we care about free-living heterotrophic stramenopiles? Heterotrophic stramenopiles are among the most abundant heterotrophs in nearly every habitat, likely playing vital roles in every ecosystem. However, our limited understanding of their life strategies hinders our assessment of their ecological importance and evolutionary impacts. By delving into their cell biology, we can unravel transitions to autotrophy, osmotrophy, and parasitism. Furthermore, as our knowledge grows, we can better integrate these organisms into complex food webs, illuminating their ecological significance.

Apart from ochrophytes, all other photosynthetic eukaryotes branch sister to extremely derived heterotrophs. Thus, stramenopiles are possibly among the best candidates to study the entire evolutionary journey of the plastid—from its acquisition, genomic and metabolic assimilation, to its potential loss. The next steps towards better understanding the process of higher-order endosymbioses would be to establish model heterotrophic stramenopiles to determine which endogenous pathways might be recruited to support an incoming plastid. Whatever the future holds, a better understanding of heterotrophic stramenopiles will help determine their pivotal roles in ecosystems and their evolutionary dynamics.

## Data Availability

Data sharing is not applicable to this article as no datasets were generated or analyzed during the current study.
