# Correction to: Integrated overview of stramenopile ecology, taxonomy, and heterotrophic origin

**DOI:** 10.1093/ismejo/wrae254

**Published:** 2025-04-15

This is a correction to: Dagmar Jirsová, Jeremy G Wideman, Integrated overview of stramenopile ecology, taxonomy, and heterotrophic origin, *The ISME Journal*, Volume 18, Issue 1, January 2024, wrae150, https://doi.org/10.1093/ismejo/wrae150

Funding from the Gordon and Betty Moore Foundation was incorrectly given as GBMF9201. This has been corrected to read GBMF10600.

